# Relative importance of gene effects for nitrogen-use efficiency in popcorn

**DOI:** 10.1371/journal.pone.0222726

**Published:** 2019-09-26

**Authors:** Adriano dos Santos, Antônio Teixeira do Amaral Júnior, Roberto Fritsche-Neto, Samuel Henrique Kamphorst, Fernando Rafael Alves Ferreira, José Francisco Teixeira do Amaral, Janieli Maganha Silva Vivas, Pedro Henrique Araújo Diniz Santos, Valter Jário de Lima, Shahid Khan, Kátia Fabiane Medeiros Schmitt, Jhean Torres Leite, Divino Rosa dos Santos Junior, Rosimeire Barboza Bispo, Talles de Oliveira Santos, Uéliton Alves de Oliveira, Lauro José Moreira Guimarães, Oscar Rodriguez

**Affiliations:** 1 Laboratório de Melhoramento Genético Vegetal, Centro de Ciências e Tecnologias Agropecuárias, Universidade Estadual do Norte Fluminense Darcy Ribeiro (UENF), Campos dos Goytacazes, RJ, Brazil; 2 Departamento de Genética, Escola Superior de Agricultura Luiz de Queiroz (ESALQ), Piracicaba, SP, Brazil; 3 Departamento de Engenharia Rural, Centro de Ciências Agrárias e Engenharias, Universidade Federal do Espírito Santo (UFES), Alegre, ES, Brazil; 4 Empresa Brasileira de Pesquisa Agropecuária (EMBRAPA), Centro Nacional de Pesquisa de Milho e Sorgo, Sete Lagoas, MG, Brazil; 5 Department of Agronomy and Horticulture, University of Nebraska, Nebraska, United States of America; Swedish University of Agricultural Sciences, SWEDEN

## Abstract

The objective of this study was to evaluate the effects of additive and non-additive genes on the efficiency of nitrogen (N) use and N responsiveness in inbred popcorn lines. The parents, hybrids and reciprocal crosses were evaluated in a 10x10 triple lattice design at two sites and two levels of N availability. To establish different N levels in the two experiments, fertilization was carried out at sowing, according to soil analysis reports. However, for the experiments with ideal nitrogen availability, N was sidedressed according to the crop requirement, whereas for the N-poor experiments sidedressing consisted of 30% of that applied in the N-rich environment. Two indices were evaluated, the Harmonic Mean of the Relative Performance (HMRP) and Agronomic Efficiency under Low Nitrogen Availability (AELN), both based on grain yield at both N levels. Both additive and non-additive gene effects were important for selection for N-use efficiency. Moreover, there was allelic complementarity between the lines and a reciprocal effect for N-use efficiency, indicating the importance of the choice of the parents used as male or female. The best hybrids were obtained from inbred popcorn lines with contrasting N-use efficiency and N responsiveness.

## Introduction

Maize is one of the most important agricultural commodities at all over the world and its production consumes almost one-fifth of all nitrogen produced. In this context, excessive applications of N fertilizers may harm the environment, causing for example soil acidification and water and air pollution [1,2]. Since the plants can take only up 30 to 40% of the applied N. Thus, over 60% of the N in the soil generally is lost by leaching, surface runoff, denitrification, volatilization, and microbial consumption.

This is an indication that more research is needed to increase maize yields with lower environmental impact. From this perspective, one option is to develop the N-use efficient (NUE) cultivars. It is well known that nitrogen use efficient is available for the genotype keeps or increases its grain quantity when low N availability occurs [3]. The Nitrogen responsiveness can be observed when a given genotype obtains higher average grain yield in the environment with good N availability than in the environment with nitrogen stress. During previous study, Kant et al. [4] estimated that a 1% increase in NUE of crops could save US$ 1.1 billion annually. Therefore, the development of NUE cultivars is required to minimize losses of applied N as well as to decrease environmental pollution, reduce inputs and consequently, save production costs.

It is noteworthy that, in spite of all breeding activities, the maize germplasm still has wide genetic variability for N-use efficiency that has not been exploited [4,5]. However, the development of NUE maize cultivars is a great challenge due to the genetic complexity and strong interaction with the environment. Thus, information on the genetic control of this trait, at contrasting levels of N availability, would be useful for crop researchers to choose the breeding method for obtaining NUE efficient cultivars.

In this context, previous studies investigated the genetic control in maize, to identify genomic regions and candidate genes related to N-use efficiency [1,6–8]. On the other hand, the magnitude of additive and non-additive effects on the trait control is not yet well understood.

Considering all these points, a diallel analysis is a potential tool for the identification of desirable parents with information about the nature and magnitude of the genetic effects controlling the trait, since the general combining ability (GCA), as is well-known, provides information about the performance of additive genes, while the specific combining ability (SCC) refers to dominant and epistatic genes. Diallel analysis has been used in studies on the inheritance and genetic control of important traits for a number of crop species [9–12].

Given the above, the goals of the present study were to investigate the genetic effects and the inheritance controlling the use efficiency and responsiveness of nitrogen in popcorn, allowing to analyze the relative importance of additive, non-additive and reciprocal effects in the control genetic characteristics.

## Material and methods

### Experimental conditions and genotypes

**Fig 1:**
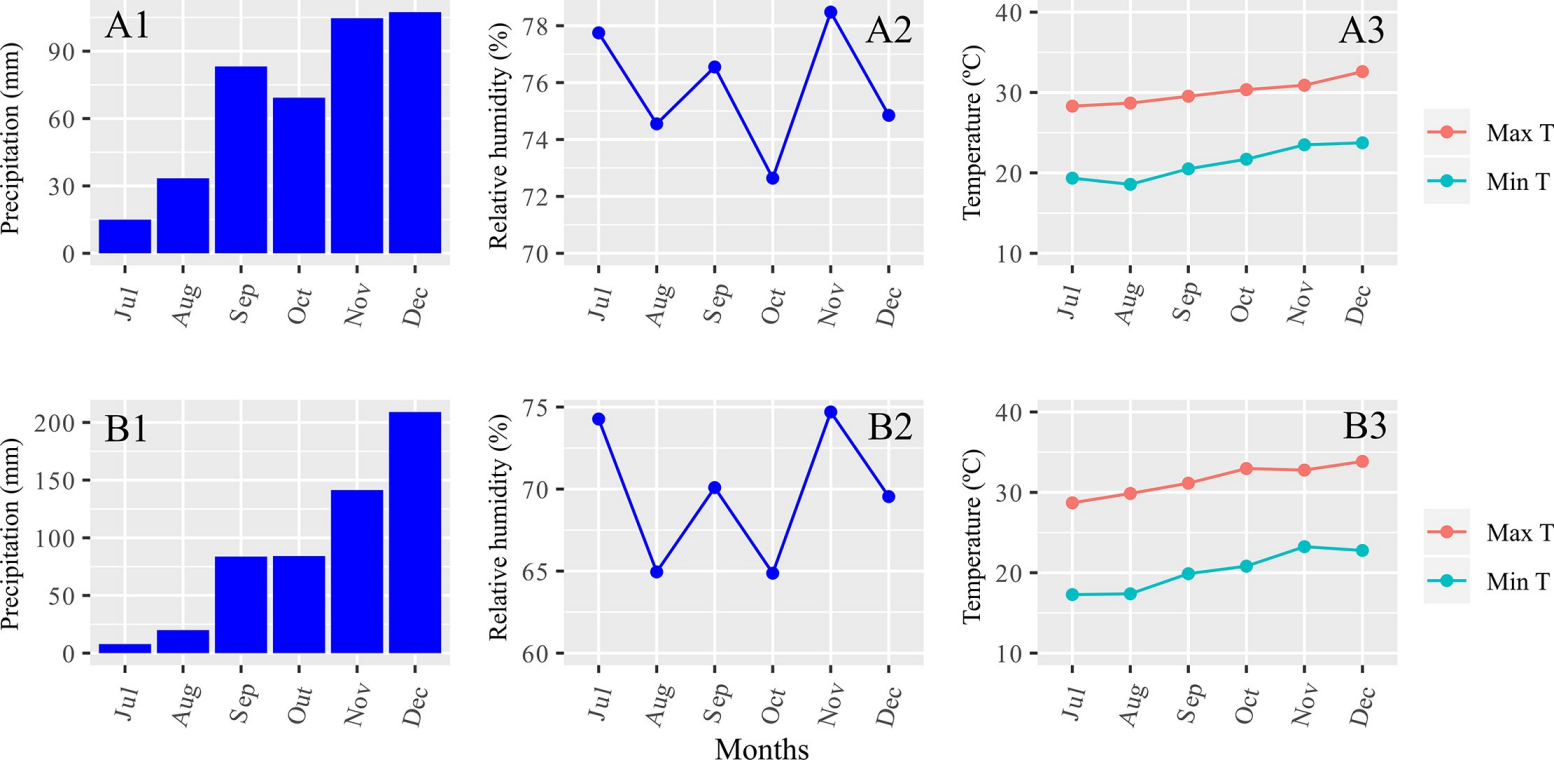
Pluviometric precipitation, minimum and maximum temperature, relative humidity during the experimental development. Campos dos Goytacazes (A1, A2 e A3), Itaocara (B1, B2, B3), RJ– 2015.

The experiments were carried out at two levels of N availability, low and optimum, corresponding to 32 and 128 kg ha^-1^ nitrogen, respectively and at two sites: Campos dos Goytacazes-RJ, at the experimental station of the Colégio Estadual Agrícola Antônio Sarlo (21° 42' 48" S, 41° 20' 38" W; 14 m asl) and at 60 m altitude, with an average annual temperature of 22.5° C and an average annual precipitation of 1041 mm (Fig 1) and soil classified as Ultisol Yellow Distrocoeso and at the Experimental Station of Itaocara-RJ (21° 38' 50" S, 42° 03' 46" W; 58 m asl), representing, respectively, the North and Northwestern regions of the State of Rio de Janeiro. The climate in both environments is tropical humid (Aw), according to the Köppen classification and soil classified as Red-Yellow Latosol. Soil chemical properties listed in S1 Table. In both experiments there was supplementary irrigation, in which the water slides were applied according to the water requirement of the crop.

The genotypes were derived from crosses in a complete diallel mating design with reciprocal crosses, among 10 S_7_ lines having contrasting N use (S2 Table). The lines L61, L75 and L80 were previously classified as inefficient in N use and nonresponsive to N (INR), lines P2, P6 and P7 were classified as N-use efficient and N-responsive (ER) and lines L54, L59, L76, and L77 were classified as intermediate in N-use efficiency and N responsiveness [13]. Thus, 45 hybrid combinations and their respective reciprocals were obtained, resulting in a total of 90 hybrid combinations.

These 90 hybrid combinations were evaluated, together with the 10 S_**7**_ parents, in a 10x10 triple lattice design in which each experimental unit consisted of one 4.2 m row with a row spacing of 0.60 m and plant spacing of 0.25 m. Two seeds per hill were sown and 21 days after emergence, thinned to one plant per hill, resulting in a stand of 18 plants per plot. Harvesting was done manually in December 2015. Minimal set data (S3 Table) is being shown in support information.

### Nitrogen availability

In order to distinguish the N level of the experiments, the following strategy was adopted: for experiments with optimal N availability, fertilization was applied at sowing according to the soil analysis, consisting of 32 kg N ha^-1^, and side dressing, for N supplementation, was performed with 128 kg N ha^-1^, split in two applications, in the stages V4 and V6 respectively, using urea as N source. For the experiments with low N availability (LN), fertilization at planting was similar to that of the experiment with optimal availability (HN) and side dressing consisted of only 30% of that applied in the environment with optimal N supply. The sowing fertilization of both nitrogen availabilities (down and ideal) constituted of the 32 kg N ha^-1^ N, 85 kg P_2_O_5_ ha^-1^ and 0 kg K_2_O ha^-1^ in Campos dos Goytacazes and Itaocara, RJ.

### Classification of the genotypes as to NUE

At harvest, the grain yield of each plot was estimated, adjusted to 13% moisture and extrapolated to kg ha^-1^. Two indices based on grain yield (kg ha^-1^) were estimated to quantify N-use efficiency of each genotype at low and high N availability. Initially, the index proposed by Wu et al. [14] was estimated, and later modified for the agronomic efficiency (AELN), according to the expression (01):

$$AELN_{ij}=\frac{GY_{\left(LN\right)}{}_{{}_{ij}}}{GY_{\left(HN\right)}{}_{{}_{ij}}}.GY_{\left(LN\right)}{}_{{}_{ij}}$$ (01)

where AELN_ij_ is the agronomic efficiency at low N availability of line *i* in replication *j*; GY_(HN)lj_ is the grain yield under optimal N availability (phenotypic value) of line *i* in replication *j*; GY_(LN)ir_ is the grain yield at low N availability (phenotypic value) of line *i* in replication *j*.

Then the Harmonic Mean of the Relative Performance (HMRP) was estimated according to the expression (02) [15]:

$$HMRP_{i}{}_{j}=\frac{2}{{\left(\frac{GY_{(HN)ij}}{{\bar{X}}_{\left(HN\right)}}\right)}^{-1}+{\left(\frac{GY_{(LN)ij}}{{\bar{X}}_{\left(LN\right)}}\right)}^{-1}}$$ (02)

where, *HMRP*_*ij*_ is the Harmonic Mean of Relative Performance of Genotype Values of line *i* in replication *j*; *GY*_*(HN)i*_ is the grain yield in the environment with ideal N availability (phenotypic value) of line *i* in the replication *j*; *GY*_*(LN)i*_ is grain yield in the environment of low N availability (phenotypic value) of line *i* in replication *j*; ${\bar{X}}_{\left(HN\right)}$ is the overall mean of the high-N environment; ${\bar{X}}_{\left(LN\right)}$ is the overall mean of the low-N environment.

### Statistical analysis

The combining ability was analyzed based on Method I of Griffing [16], in which the p^2^ combinations are evaluated, including parents, hybrids and their reciprocals. The genotype effects were considered fixed, based on the expression (03):

$$Y_{ij}=m+g_{i}+g_{j}+s_{ij}+r_{ij}+e_{k}+ge_{ik}+ge_{jk}+se_{ijk}+re_{ijk}+\varepsilon_{ijk}$$ (03)

where: *Y*_*ij*_: is mean value of hybrid combination *ij*; m: the general constant inherent to the observations; *g*_*i*_ and *g*_*j*_: are effects of the general combining ability of the *ij* order parents, respectively; *s*_*ij*_: is the effect of specific combining ability for crosses between the ith and jth order genders; *r*_*ij*_: is the reciprocal effect that measures the differences provided by parent *i*, or *j*, when used as male or female in cross *ij*; *e*_*k*_: is the effect of environment k; *ge*_*ik*_ and *ge*_*jk*_: are effects of the interaction between the general combining ability of the parents *i* and *j*, respectively, with environment k; *se*_*ijk*_: is the effect of the interaction between the specific combining ability between the parents *i* and *j* at environment k; *re*_*ijk*_: is the interaction between the reciprocal effect and environment *k*; *ɛ*_*ijk*_: is the mean experimental error associated with observation of order *ij*, with NID ~ (0, σ^2^).

The mean dominance degree (*MDD*) was estimated by the expression: *MDD* = 2σ^2^_CGC_/(2σ^2^_CGC_ + σ^2^_CEC_), proposed by Baker [17], where 2σ^2^_GCA_ is the quadratic form (analogous to the components of variance, but referring to the fixed effect) derived from the effect of the mean square of the GCA and σ^2^_SCA_ is the quadratic form of the effects of the SCA. The analyses were performed using SAS macro “Diallel-SAS05”, as described by Zhang et al. [18].

## Results

The genotype effect was highly significant (p < 0.01) for all traits analyzed, evidencing the existence of genetic variability for these traits in the studied population. This indicates the possibility of success in the selection of superior genotypes for N-use efficiency, with this base population in a breeding program. The same result can be observed for general and specific combining ability and for the reciprocal effect (REC), in which all traits were significant at (p < 0.01) (Table 1).

**Table 1:** Combined diallel analysis for agronomic efficiency at low N availability (AELN), harmonic mean of the relative performance (HMRP) and grain yield (GY) in popcorn genotypes.

Source of Variation	DF	AELN	HMRP	GY	
				Campos	Itaocara
Replication (Environment)	4	432448.6	0.00116	20845.00	79168.7
Environment (E)	1	81968638.9	0.00157	4294487.2**	79222167.2**
Genotype (G)	99	3328381.7**	0.5299**	975235.9**	2314001.2**
GCA	9	6892761.26**	1.4393**	8713567.9**	10746365.92**
SCA	45	3584582.2**	0.6213**	3213312.2**	8446332.37**
Reciprocal (REC)	45	2359305.35**	0.2567**	1526251.4**	3016353.49**
Maternal (M)	9	4676632.91**	0.9409**	5527482.9**	6873211.29**
Non-maternal (NM)	36	1779973.46**	0.0856**	525943.59**	2052139.04**
G X E	99	1633930.3**	0.1190**	968981.02**	965416.2**
GCA x E	9	2329523.58**	0.3143**	614243.65*	318340.39**
SCA x E	45	1277566.42**	0.0921**	160757.96*	895005.47**
REC x E	45	1851175.61**	0.1068**	167055.06*	1165242.15**
M x E	9	2613974.11**	0.1853**	226979.11*	1520772.94**
NM x E	36	1660475.98**	0.0872**	152074.04*	1076359.45**
Error	396	123510.5	0.00375	19451.0	51665.8
*MDD*	-	0.79	0.82	0.84	0.72

*: significant at the 5% probability level by the F test

**: significant at the 1% probability level by the F test. GY: kg ha^-1^.

For the indices AELN and HMRP, the interactions GCA x E, SCA x E, REC x E, M x E, and NM x E were significant. In this case, the environment (E) consisted of sites, indicating that the effects observed in each environment were not consistent, in other words, a parent with high additive effects at one site may not have the same superiority at another. The effects of the interactions GCA x E, SCA x E, REC x E, M x E, and NM x E were also significant for grain yield, although it is worth mentioning that in this study, the environments were represented by the different N availability levels. In this way, the performance of parents (ĝ_i_) and crosses (ŝ_ij_) should be observed in each environment, to ensure the selection or breeding of genotypes for specific conditions.

The magnitude of the mean degree of dominance (MDD) ranged from 0.72 to 0.84 for grain yield in Itaocara and for AELN. This result can be corroborated by the higher values of the average squares of the overall combining ability over specific capacity.

Estimates of the GCA effects provide information about the number of additive gene effects. Thus, the parents with highest ĝ_i_ values are the most promising for breeding programs selecting for N-use efficiency. In this way, the parents L59, L61, P2, P6, and P7 stood out in the different environments and in the mean of the environments, and although the GCA x E interaction was significant, the ĝ_i_ estimates of these parents were positive for AELN and HMRP (Table 2).

**Table 2:** Effect of the general combining ability (ĝ_i_) on the agronomic efficiency at low N availability (AELN), harmonic mean of the relative performance of the predicted genetic values (HMRP) and agronomic efficiency of nitrogen (AELN) and standard deviation (SD) of popcorn genotypes evaluated at two locations in the 2015/2016 growing season.

Parent		AELN			HMRP	
	Campos	Itaocara	Mean	Campos	Itaocara	Mean
L54	-279.94	166.09	-56.93	-0.15	0.00	-0.08
L59	538.17	6.58	272.37	0.22	0.01	0.11
L61	113.06	83.94	98.50	0.15	0.02	0.09
L75	-273.03	-482.99	-378.01	-0.19	-0.18	-0.18
L76	-432.56	-170.31	-301.44	-0.06	0.00	-0.03
L77	-127.71	111.77	-7.97	0.00	0.05	0.03
L80	-220.12	-276.72	-248.42	-0.18	-0.14	-0.16
P2	340.41	177.58	259.00	0.08	0.06	0.07
P6	188.94	121.63	155.29	0.01	0.04	0.02
P7	152.79	262.44	207.61	0.12	0.14	0.13
SD	35.85	49.38	-	0,0077	0.0072	-

About grain yield, the parents P7, L61 and L59, in Campos dos Goytacazes, and P2, P6 and P7, in Itaocara, had the biggest ĝi effects in low and high N levels, presenting the efficiency of these parents under low N availability and responsivity. Parent P7 had both high N responsiveness and N-use efficiency, and parent P2 was also highly NUE (Table 3).

**Table 3:** Effect of the general combining ability (ĝ_i_) on popcorn grain yield and standard deviation (SD) evaluated at high and low N availability in Campos dos Goytacazes and Itaocara, in the 2015/2016 growing season.

Parents	Campos dos Goytacazes		Itaocara		Mean
	High N	Low N	High N	Low N	
L54	-312.80	-277.46	-124.50	76.00	-159.69
L59	377.37	443.74	72.15	8.11	225.34
L61	325.92	227.87	59.54	64.47	169.45
L75	-408.94	-318.43	-620.75	-537.38	-471.37
L76	91.53	-229.82	39.23	-51.28	-37.58
L77	50.29	-39.24	131.33	140.25	70.66
L80	-388.17	-291.73	-436.39	-402.17	-379.62
P2	67.48	213.89	161.41	197.79	160.14
P6	50.29	76.69	177.53	124.81	82.19
P7	247.59	194.48	540.46	379.40	340.48
SD	15.00	18.98	25.08	30.25	-

It should be noted that parent L61, considered N-use inefficient, proved NUE at both locations and N levels, and it can be inferred that for an accurate selection of NUE genotypes, apart from selection *per se*, the estimates of ĝ_*i*_ and ŝ_*ij*_ should be used, since selection *per se* of a genotype provides no information about the GCA and SCA.

An ideal parent for breeding should be highly N responsive and highly NUE under low N availability. The hybrid combinations L59 x L80, L61 x L77, L54 x L76, L59 x P2, and L77 x P6 had the highest ŝ_*ij*_ effects on AELN (Table 4). Interestingly, parents L77, L54 and L80 had negative ĝ_*i*_ estimates, allowing to conclude that the ŝ_*ij*_ effects of these crosses expressed allelic complementation. These results from the difference in allelic frequency between the parents (divergence) and from dominance effects [10], represented by the parents L59, L61 and P6, which had a relatively high mean ĝ_*i*_ value for AELN.

**Table 4:** Effect of specific combining ability (ŝ_ij_) on the agronomic efficiency under low nitrogen availability (AELN) and standard deviation (SD) of popcorn genotypes evaluated in Campos dos Goytacazes and Itaocara, in the 2015/2016 growing season.

Genotypes	Campos	Itaocara	Mean	Genotypes	Campos	Itaocara	Mean
L54 x L59	249.37	711.18	480.27	L61 x P7	470.62	-106.23	182.20
L54 x L61	23.89	278.16	151.03	L75 x L76	-340.72	-315.20	-327.96
L54 x L75	-56.63	-270.13	-163.38	L75 x L77	451.80	-333.44	59.18
L54 x L76	950.07	441.60	695.83	L75 x L80	275.45	492.03	383.74
L54 x L77	-118.96	425.62	153.33	L75 x P2	-510.80	921.06	205.13
L54 x L80	212.17	466.16	339.16	L75 x P6	65.14	370.36	217.75
L54 x P2	-217.54	110.02	-53.76	L75 x P7	176.41	170.45	173.43
L54 x P6	-271.23	130.97	-70.13	L76 x L77	-85.88	623.73	268.93
L54 x P7	-198.80	-142.62	-170.71	L76 x L80	-119.38	-121.78	-120.58
L59 x L61	-63.37	-62.27	-62.82	L76 x P2	-44.33	449.93	202.80
L59 x L75	20.58	-409.58	-194.50	L76 x P6	-128.75	542.85	207.05
L59 x L76	-136.30	-74.83	-105.57	L76 x P7	481.29	-132.90	174.20
L59 x L77	28.41	-51.79	-11.69	L77 x L80	-167.20	-234.81	-201.01
L59 x L80	232.52	1934.96	1083.74	L77 x P2	332.20	-609.29	-138.54
L59 x P2	923.92	337.99	630.95	L77 x P6	198.24	885.91	542.07
L59 x P6	1035.66	-115.77	459.94	L77 x P7	-52.08	584.25	266.09
L59 x P7	-244.07	-194.20	-219.14	L80 x P2	276.37	-595.94	-159.79
L61 x L75	485.11	393.01	439.06	L80 x P6	-298.78	-1234.48	-766.63
L61 x L76	156.35	350.78	253.56	L80 x P7	257.77	82.27	170.02
L61 x L77	616.33	883.64	749.99	P2 x P6	609.47	190.58	400.03
L61 x L80	-244.58	-10.05	-127.31	P2 x P7	-410.53	-415.25	-412.89
L61 x P2	131.00	-206.48	-37.74	P6 x P7	-245.96	-880.53	-563.25
L61 x P6	-198.16	323.42	62.63	-	-	-	-
SD	108.21	149.05	-	-	108.21	149.05	-

Similarly to AELN, used as a *per se* index of simultaneous selection, HMRP is an index that takes high-yielding genotypes in all environments into account and consequently enables the selection of productive genotypes under optimal and low N availability. The best crosses with the highest mean ŝ_*ij*_ effects were L54 x L76, L59 x L77, L77 x P7, L61 x L77, and L59 x P7 (Table 5).

**Table 5:** Effect of specific combining ability (ŝ_*ij*_) for the harmonic mean of relative performance (HMRP) and standard deviation (SD), of popcorn genotypes evaluated in Campos dos Goytacazes and Itaocara, in the 2015/2016 growing season.

Genotypes	Campos	Itaocara	Mean	Genotypes	Campos	Itaocara	Mean
L54 x L59	-0.146	0.133	-0.006	L61 x P7	0.030	-0.061	-0.015
L54 x L61	0.068	0.235	0.152	L75 x L76	-0.248	-0.104	-0.176
L54 x L75	0.053	-0.043	0.005	L75 x L77	0.073	0.019	0.046
L54 x L76	0.368	0.162	0.265	L75 x L80	0.033	0.042	0.038
L54 x L77	0.037	0.032	0.035	L75 x P2	-0.072	0.026	-0.023
L54 x L80	0.126	0.202	0.164	L75 x P6	0.098	0.224	0.161
L54 x P2	-0.049	0.156	0.054	L75 x P7	-0.021	0.070	0.024
L54 x P6	-0.022	0.139	0.059	L76 x L77	0.140	0.034	0.087
L54 x P7	-0.082	-0.270	-0.176	L76 x L80	0.083	0.145	0.114
L59 x L61	0.194	-0.085	0.055	L76 x P2	-0.001	0.239	0.119
L59 x L75	0.214	0.071	0.142	L76 x P6	0.070	0.077	0.073
L59 x L76	0.149	-0.058	0.046	L76 x P7	-0.009	0.097	0.044
L59 x L77	0.084	0.371	0.228	L77 x L80	-0.239	-0.265	-0.252
L59 x L80	0.109	0.051	0.080	L77 x P2	0.229	0.004	0.117
L59 x P2	-0.022	0.095	0.036	L77 x P6	0.086	0.204	0.145
L59 x P6	0.115	-0.065	0.025	L77 x P7	0.017	0.395	0.206
L59 x P7	0.242	0.105	0.174	L80 x P2	-0.060	-0.143	-0.101
L61 x L75	0.123	0.171	0.147	L80 x P6	0.039	-0.063	-0.012
L61 x L76	0.095	0.246	0.171	L80 x P7	0.090	0.212	0.151
L61 x L77	0.309	0.084	0.197	P2 x P6	0.089	0.068	0.078
L61 x L80	-0.081	0.127	0.023	P2 x P7	0.032	0.006	0.019
L61 x P2	0.130	0.054	0.092	P6 x P7	-0.213	-0.207	-0.210
L61 x P6	0.065	-0.072	-0.003	-	-	-	-
SD	0.023	0.021	-	-	0.023	0.021	-

In Campos dos Goytacazes, the highest ŝ_*ij*_ effects were observed in the combinations L54 x L76, L61 x L77, L59 x P7, and L77 x P2. The ŝ_*ij*_ effect of these crosses was high in the presence and absence of nitrogen, showing that these genotypes were NUE and N responsive. In Itaocara, we can observe that the highest values of ŝ_*ij*_ were for the combinations L59 x L77, L77 x P7, L61 x L76, L54 x L61 and L75 x P6, both for environments with high and low nitrogen availability (Tables 6 and 7). The ŝ_ij_ effect for the hybrids L54 x L76, L54 x L80 and others indicated deviations from what was expected, based on the GCA of the parents L54 and L76, evidencing non-additive gene effects [9].

**Table 6:** Effect of the specific combining ability (ŝ_*ij*_) on grain yield and standard deviation (SD) of popcorn genotypes evaluated under optimal and low N availability in Campos dos Goytacazes, in the 2015/2016 growing season.

Genotypes	High N	Low N	Mean	Genotypes	High N	Low N	Mean
L54 x L59	-439.18	-96.93	-268.05	L61 x P7	-85.11	198.90	56.90
L54 x L61	174.74	88.12	131.43	L75 x L76	-549.55	-412.83	-481.19
L54 x L75	158.45	41.23	99.84	L75 x L77	28.60	242.75	135.67
L54 x L76	482.81	794.32	638.57	L75 x L80	9.32	130.38	69.85
L54 x L77	198.64	-6.55	96.04	L75 x P2	-3.50	-260.98	-132.24
L54 x L80	262.32	220.68	241.50	L75 x P6	219.26	146.86	183.06
L54 x P2	-40.78	-132.90	-86.84	L75 x P7	-52.67	24.83	-13.92
L54 x P6	24.24	-113.95	-44.85	L76 x L77	453.40	120.55	286.98
L54 x P7	-133.61	-167.89	-150.75	L76 x L80	271.33	54.13	162.73
L59 x L61	500.82	216.79	358.81	L76 x P2	-89.93	-1.71	-45.82
L59 x L75	486.32	276.45	381.39	L76 x P6	192.22	42.77	117.50
L59 x L76	492.05	123.95	308.00	L76 x P7	-106.51	143.63	18.56
L59 x L77	134.87	127.95	131.41	L77 x L80	-545.30	-350.21	-447.76
L59 x L80	161.35	229.91	195.63	L77 x P2	419.23	405.13	412.18
L59 x P2	-283.59	271.57	-6.01	L77 x P6	117.80	182.37	150.09
L59 x P6	14.41	468.29	241.35	L77 x P7	73.58	0.93	37.25
L59 x P7	672.22	212.14	442.18	L80 x P2	-200.80	15.04	-92.88
L61 x L75	143.10	319.07	231.08	L80 x P6	172.60	-52.10	60.25
L61 x L76	163.38	172.23	167.80	L80 x P7	106.87	203.56	155.21
L61 x L77	535.05	593.55	564.30	P2 x P6	48.91	310.76	179.83
L61 x L80	-99.24	-184.64	-141.94	P2 x P7	194.57	-100.69	46.94
L61 x P2	291.28	202.51	246.90	P6 x P7	-475.14	-337.03	-406.08
L61 x P6	266.56	5.92	136.24	-	-	-	-
SD	45.27	57.31	-	-	45.27	57.31	-

**Table 7:** Effect of specific combining ability (ŝ_*ij*_) on grain yield and standard deviation (SD) of popcorn genotypes evaluated under high and low N availability in Itaocara, in the 2015/2016 growing season.

Genotypes	High N	Low N	Mean	Genotypes	High N	Low N	Mean
L54 x L59	234.50	561.81	398.15	L61 x P7	-342.47	-141.84	-242.16
L54 x L61	915.33	587.39	751.36	L75 x L76	-381.93	-324.80	-353.36
L54 x L75	-56.23	-188.20	-122.21	L75 x L77	309.60	-106.43	101.58
L54 x L76	532.24	497.24	514.74	L75 x L80	-108.80	285.25	88.23
L54 x L77	-50.87	237.51	93.32	L75 x P2	-167.46	384.05	108.30
L54 x L80	723.42	584.24	653.83	L75 x P6	784.16	608.21	696.18
L54 x P2	671.67	353.94	512.81	L75 x P7	153.04	228.96	191.00
L54 x P6	638.77	304.21	471.49	L76 x L77	-60.97	304.66	121.84
L54 x P7	-1090.98	-613.58	-852.28	L76 x L80	779.83	233.05	506.44
L59 x L61	-238.05	-200.48	-219.26	L76 x P2	931.15	650.00	790.58
L59 x L75	566.63	-29.82	268.41	L76 x P6	39.78	377.66	208.72
L59 x L76	-315.33	-133.00	-224.16	L76 x P7	607.42	120.44	363.93
L59 x L77	1789.23	704.15	1246.69	L77 x L80	-1212.17	-611.20	-911.68
L59 x L80	-520.37	806.09	142.86	L77 x P2	406.81	-248.10	79.36
L59 x P2	164.18	346.55	255.36	L77 x P6	461.98	765.02	613.50
L59 x P6	-449.36	-137.63	-293.49	L77 x P7	1498.55	1027.79	1263.17
L59 x P7	561.21	134.03	347.62	L80 x P2	-523.40	-489.22	-506.31
L61 x L75	751.14	468.34	609.74	L80 x P6	820.40	-668.81	75.79
L61 x L76	984.01	625.16	804.58	L80 x P7	941.77	455.69	698.73
L61 x L77	13.41	504.66	259.03	P2 x P6	98.97	230.12	164.55
L61 x L80	478.30	267.58	372.94	P2 x P7	252.85	-154.54	49.16
L61 x P2	262.81	41.85	152.33	P6 x P7	-647.84	-742.26	-695.05
L61 x P6	-237.02	-66.42	-151.72	-	-	-	-
SD	75.70	91.32	-	-	75.70	91.32	-

## Discussion

The effects of GCA (ĝ_i_) on the additive effects or frequency of favorable alleles for N-use efficiency and the SCA (ŝ_ij_), which are related to the non-additive gene effects (dominance and epistasis) or effects of intragenic and intergenic complementation. The significance for the effects of ĝ_i_ indicate that at least one parent differs from the others in terms of the number of favorable additive effect genes. The significance for ŝ_ij_ indicate the existence of allelic complementation among the parents with loci containing some degree of dominance.

In general, for grain production in the maize crop, the non-additive genetic effects such as the dominance are prevalent, for example [19]. The results show that the additive effect presented bigger contribution to the expression of characters. As the additive effects are essential in the predictability of genetic expression of quantitative characters, this result makes it possible to obtain superior populations for NUE, using intrapopulational breeding methods.

It should also be noted that the significance of REC influences the estimation of genetic effects. The reciprocal effect can be attributed to cytoplasmic genetic factors or the interaction between nuclear genes and the effects of cytoplasmic genes [20]. This shows how important an appropriate choice of the female parent is. This reciprocal effect may be related to processes of N metabolism, e.g., photosynthesis, in which extra-nuclear genes are important for the formation of the photosynthetic apparatus of the chloroplasts [20]. Thus, it is advisable to use NUE genotypes as female parents in the crossing blocks.

The observed interactions between general, specific combining ability and reciprocal effect with environments for all variables, allows us to suggest that the selection of NUE genotypes, based on grain yield, should be environment-specific rather than based on the mean performance, since the alleles that control the trait expression under low nutrient supply are partially different from those that control the same trait under ideal nutrient supply [21]. This implies that grain yield at low N availability involves not only the genes expressed under ideal N availability, but that other genes are also expressed or silenced [22].

This difference in gene expression was clearly observed in the studies of Gallais and Hirel [7], Zheng et al. [23] and Liu et al. [22], describing a differentiated expression of genetic variability in maize at low and optimal N levels, because different Quantitative Trait Loci (QTLs) were found for these environmental conditions. This difference in QTL number between the two N levels may reflect differences in the sensitivity of the studied variables and differences in the genotype responses to environments [24]. Therefore, these QTLs may be useful to raise the productivity of new genotypes, by combining yield-related QTLs expressed at low N availability levels with QTLs for N-use efficiency identified in low-N environments.

Based on the MDD, conclusions can be drawn about the type of genetic action related to the genetic control of the trait. Mean degrees of dominance close to unity suggest that the SCA effect is not predominant and that the hybrid performance can be predicted from the mean ĝ_i_ values of the parents [16]. Consequently, additive genetic variance is more important than the effects of dominance for N-use efficiency in popcorn. Nevertheless, non-additive effects should not be disregarded, since they also have a certain influence on trait expression. This implies that artificial selection for plants with the desirable traits will possibly result in progenies with superior performance [25], because in case of additive gene effects, the mean of the parents is equal to the mean of F_1_, F_2_ and so on [10]. This result confirms literature reports, citing additive effects as the most relevant for N-use efficiency components [26,27].

One of the reasons that may be related to the superiority of the expression of the gene effects responsible for the efficiency in nitrogen use is the choice of the parents in the crossing blocks, since they can be used as inbred lines, thus presenting a narrow genetic basis or even open pollinated genotypes, which exhibit a broad genetic basis [28]. Therefore, it is evident that each parent may contribute differently to the crossing blocks, since they will have more or less frequency of favorable alleles, providing contrasting results in relation to the predominant type of gene action for a given trait. We can also mention the genetic distance between parents, because this distancing interferes with the genetic effects, as it is one of the components used for the estimation of heterosis. Therefore, it is evident the importance of obtaining information of the genetic effects related to nitrogen use efficiency in popcorn.

However, caution should be exercised when selecting for N-use efficiency based on grain yield only, since non-additive effects are known as relevant for grain yield control at the different N levels [29,30]. Therefore, both additive and non-additive effects should be taken into consideration. In this way, different methods for the selection of parents of new cultivars can be explored. However, among the breeding methods, interpopulation reciprocal recurrent selection is highlighted as a particularly promising option, for exploiting both the additive and dominance genetic variance.

A similar reaction was observed between the best crosses for AELN and HMRP. This finding may be related to the fact that the AELN index is mainly used for the selection of high-yielding genotypes under low N availability [13] whereas, according to Resende [14], index HMRP leads to simultaneous selection for stability and adaptability. In other words, it can be used for the selection of genotypes with above-average yield means under ideal and stress conditions.

The hybrids L61 x L76 and L61 x L77 combine a parent with intermediate N-use efficiency and N responsiveness (L76 and L77) with an inefficient one (L61), while hybrid L80 x P7 combines an efficient (P7) with an inefficient parent (L80) (Tables 6 and 7). These results demonstrate the occurrence of allelic complementation among these parents. Two parents with high ĝ_*i*_ do not always result in a higher ŝ_*ij*_, as observed for cross L61 x P7, since this depends on satisfactory allelic complementation and dominance [10]. It is rather uncommon to observe two parents with low ĝ_*i*_ estimates resulting in a high ŝ_*ij*_ estimate. However, it should be noted that the ŝ_*ij*_ estimates of the hybrids L54 x L76 and L54 x L80 were high in Campos dos Goytacazes and Itaocara, respectively.

Among the evaluated parents, P7 and L59 can be considered very efficient *per se*. Since an ideal parent should have a high ĝ_*i*_ effect and participate in crosses with high ŝ_*ij*_, these parents can be considered promising in terms of N responsiveness, since both have high ĝ_*i*_ estimates and participate in the crosses with highest ŝ_*ij*_ effects in the N-rich environment. For N-use efficiency, parent P7 is the most promising, for having highest ĝ_*i*_ and participating in the cross with highest ŝ_*ij*_ in the N-poor environment.

## Conclusions

The partial dominance controls the efficiency of using N. Thus, the additive effects are the most important, but non-additive effects are relevant for the traits, as well. For proper reflexes, extrachromosomal genes may influence the expression of NUE. Based on this, the selection of female genotypes with higher NUE enables more productive hybrids. For better grain yield at low N availability, genes can be expressed or silenced. The grain yield is associated with non-additive effects at different levels of N. In this case, the selection for the trait can be made from genotypes where the average yield is above the average under ideal and stress conditions.

The interpopulation recurrent selection is the best option to explore both additive and dominance effects expressed in the studied traits. The hybrids L61 x L76 and L61 x L77 were the most efficient in the use of nitrogen, while the parents P7 and L59 were considered the most efficient among the studied and tend to generate more productive hybrids.

## Supporting information

S1 Table Soil chemical properties of experimental areas in Itaocara and Campos dos Goytacazes, in the layers 0–10 and 10–20 cm. (DOCX)

S2 Table Characterization of the diallel parents in terms of population structure, origin, climate adaptation, nitrogen use efficiency, cycle and susceptibility (S) and resistance (R) to *B. maydis* and *E. turcicum*. (DOCX)

S3 Table Minimal set data. (PDF)
