# Supplementary material for: Relative importance of gene effects for nitrogen-use efficiency in popcorn
Source: PLoS One. 2019 Sep 26;14(9):e0222726. doi: 10.1371/journal.pone.0222726 (PMC6762054; doi:10.1371/journal.pone.0222726)
Supplement: S1 Table — (DOCX) [file pone.0222726.s001.docx]

**S1 Table.** Soil chemical properties of experimental areas in Itaocara and Campos dos Goytacazes, in the layers 0-10 and 10-20 cm.

|  | Itaocara, RJ | | | | | | | | | |
| --- | --- | --- | --- | --- | --- | --- | --- | --- | --- | --- |
| Layer | pH | P | K | Ca | Mg | Al | H+Al | Na | C | OM |
|  | H_2_O | Mg dm^-3^ | ............................mmol dm^-3^............................ | | | | | | g dm^-3^ | |
| 0 – 10 cm | 5.1 | 5.0 | 3.7 | 11.9 | 8.0 | 1.2 | 18.2 | 1.1 | 8.9 | 15.3 |
| 10 – 20 cm | 4.9 | 2.0 | 1.9 | 11.9 | 7.2 | 1.8 | 17.8 | 0.6 | 9.3 | 16.0 |
|  | Campos dos Goytacazes, RJ | | | | | | | | | |
| Layer | pH | P | K | Ca | Mg | Al | H+Al | Na | C | OM |
|  | H_2_O | mg dm^-3^ | ............................mmol dm^-3^............................ | | | | | | g dm^-3^ | |
| 0 – 10 cm | 4.8 | 22.0 | 2.6 | 16.3 | 8.5 | 1.2 | 21.4 | 12.3 | 8.9 | 15.3 |
| 10 – 20 cm | 5.0 | 10.0 | 1.7 | 15.6 | 7.4 | 1.5 | 18.3 | 10.3 | 9.3 | 16.0 |
