# Supplementary material for: Relative importance of gene effects for nitrogen-use efficiency in popcorn
Source: PLoS One. 2019 Sep 26;14(9):e0222726. doi: 10.1371/journal.pone.0222726 (PMC6762054; doi:10.1371/journal.pone.0222726)
Supplement: S2 Table — (DOCX) [file pone.0222726.s002.docx]

**Table S2.** Characterization of the diallel parents in terms of population structure, origin, climate adaptation, nitrogen use efficiency, cycle and susceptibility (S) and resistance (R) to *B. maydis* and *E. turcicum*.

| **Genotypes** | **Type** | **Obtaining year** | **Origin** | **Climate adaptation** | **Institution of development** | **Cycle** | **NUE** |
| --- | --- | --- | --- | --- | --- | --- | --- |
| P2 | S_7_ | 2006 | Composto CMS-42 | Temperate/ Tropical | UEM | Early | ER |
| P6 | S_7_ | 2006 | Híbrido Zaeli | Temperate / Tropical | UEM | Middle | ER |
| P7 | S_7_ | 2006 | Híbrido Zaeli | Temperate / Tropical | UEM | Early | ER |
| L 54 | S_7_ | 2009 | Beija-flor: UFV | Temperate / Tropical | UENF | Early | Intermediate |
| L 59 | S_7_ | 2009 | Beija-flor: UFV | Temperate / Tropical | UENF | Early | Intermediate |
| L 76 | S_7_ | 2009 | Viçosa: UFV | Temperate / Tropical | UENF | Middle | Intermediate |
| L 77 | S_7_ | 2009 | Viçosa: UFV | Temperate / Tropical | UENF | Late | Intermediate |
| L 80 | S_7_ | 2009 | Viçosa: UFV | Temperate / Tropical | UENF | Late | INR |
| L 61 | S_7_ | 2009 | Ângela: EMBRAPA | Tropical | UENF | Late | INR |
| L 75 | S_7_ | 2009 | Viçosa: UFV | Temperate / Tropical | UENF | Late | INR |

INR: Inefficient in N use and nonresponsive to N; ER: N-use efficient and N-responsive.
