# Supplementary material for: Relative importance of gene effects for nitrogen-use efficiency in popcorn
Source: PLoS One. 2019 Sep 26;14(9):e0222726. doi: 10.1371/journal.pone.0222726 (PMC6762054; doi:10.1371/journal.pone.0222726)
Supplement: S3 Table — (PDF) [file pone.0222726.s003.pdf]

F	M	REP	HYBRID	YIELD	ENV
1	1	1	1	628,57	1
1	1	2	1	603,81	1
1	1	3	1	683,33	1
1	2	1	2	1574,60	1
1	2	2	2	1807,94	1
1	2	3	2	1169,84	1
1	3	1	3	2757,14	1
1	3	2	3	2526,98	1
1	3	3	3	2201,59	1
1	4	1	4	1247,62	1
1	4	2	4	925,40	1
1	4	3	4	939,68	1
1	5	1	5	2249,21	1
1	5	2	5	2400,00	1
1	5	3	5	2325,40	1
1	6	1	6	1614,29	1
1	6	2	6	1849,21	1
1	6	3	6	1817,46	1
1	7	1	7	1804,76	1
1	7	2	7	1852,38	1
1	7	3	7	1728,57	1
1	8	1	8	1311,24	1
1	8	2	8	1015,87	1
1	8	3	8	1161,90	1
1	9	1	9	1320,63	1
1	9	2	9	1320,63	1
1	9	3	9	1328,57	1
1	10	1	10	1461,90	1
1	10	2	10	1503,17	1
1	10	3	10	1550,79	1
2	1	1	11	1653,97	1
2	1	2	11	1644,44	1
2	1	3	11	1582,54	1
2	2	1	12	1052,38	1
2	2	2	12	933,33	1
2	2	3	12	915,87	1
2	3	1	13	3884,13	1
2	3	2	13	3796,83	1
2	3	3	13	3733,33	1
2	4	1	14	2146,03	1
2	4	2	14	2034,92	1
2	4	3	14	1868,25	1
2	5	1	15	2606,35	1
2	5	2	15	2649,21	1
2	5	3	15	2709,52	1
2	6	1	16	2771,43	1
2	6	2	16	2519,05	1
2	6	3	16	2765,08	1
2	7	1	17	2090,48	1

2	7	2	17	2052,38	1
2	7	3	17	1985,71	1
2	8	1	18	1838,10	1
2	8	2	18	1850,79	1
2	8	3	18	1753,97	1
2	9	1	19	2193,92	1
2	9	2	19	2200,00	1
2	9	3	19	2284,13	1
2	10	1	20	3304,76	1
2	10	2	20	3328,57	1
2	10	3	20	3241,27	1
3	1	1	21	1884,13	1
3	1	2	21	1692,06	1
3	1	3	21	1757,14	1
3	2	1	22	2566,03	1
3	2	2	22	2423,81	1
3	2	3	22	2511,11	1
3	3	1	23	697,62	1
3	3	2	23	713,49	1
3	3	3	23	729,37	1
3	4	1	24	1733,33	1
3	4	2	24	1887,30	1
3	4	3	24	1703,17	1
3	5	1	25	2376,19	1
3	5	2	25	2328,57	1
3	5	3	25	2360,32	1
3	6	1	26	3320,63	1
3	6	2	26	3217,46	1
3	6	3	26	3250,79	1
3	7	1	27	1766,67	1
3	7	2	27	1606,35	1
3	7	3	27	1838,10	1
3	8	1	28	1887,30	1
3	8	2	28	1880,95	1
3	8	3	28	1858,73	1
3	9	1	29	1828,57	1
3	9	2	29	1728,57	1
3	9	3	29	1868,25	1
3	10	1	30	2200,81	1
3	10	2	30	2273,02	1
3	10	3	30	2230,16	1
4	1	1	31	1765,08	1
4	1	2	31	1677,78	1
4	1	3	31	1763,49	1
4	2	1	32	2892,06	1
4	2	2	32	2846,03	1
4	2	3	32	2655,56	1
4	3	1	33	2390,48	1
4	3	2	33	2107,94	1
4	3	3	33	2225,40	1

4	4	1	34	715,71	1
4	4	2	34	683,73	1
4	4	3	34	681,75	1
4	5	1	35	984,13	1
4	5	2	35	1011,11	1
4	5	3	35	907,94	1
4	6	1	36	1641,27	1
4	6	2	36	1714,29	1
4	6	3	36	1819,05	1
4	7	1	37	1215,87	1
4	7	2	37	1344,44	1
4	7	3	37	1328,57	1
4	8	1	38	1042,86	1
4	8	2	38	1219,05	1
4	8	3	38	1339,68	1
4	9	1	39	1304,76	1
4	9	2	39	1114,29	1
4	9	3	39	1058,73	1
4	10	1	40	1411,11	1
4	10	2	40	1501,59	1
4	10	3	40	1622,22	1
5	1	1	41	2196,83	1
5	1	2	41	2034,92	1
5	1	3	41	2079,37	1
5	2	1	42	3071,43	1
5	2	2	42	3101,59	1
5	2	3	42	3344,44	1
5	3	1	43	2780,95	1
5	3	2	43	2693,65	1
5	3	3	43	2638,10	1
5	4	1	44	841,27	1
5	4	2	44	1328,57	1
5	4	3	44	1423,81	1
5	5	1	45	836,51	1
5	5	2	45	753,97	1
5	5	3	45	858,73	1
5	6	1	46	2384,13	1
5	6	2	46	2550,79	1
5	6	3	46	2439,68	1
5	7	1	47	1614,29	1
5	7	2	47	1812,70	1
5	7	3	47	1804,76	1
5	8	1	48	1407,94	1
5	8	2	48	1474,60	1
5	8	3	48	1534,92	1
5	9	1	49	2092,06	1
5	9	2	49	1995,24	1
5	9	3	49	2136,51	1
5	10	1	50	2090,48	1
5	10	2	50	2011,11	1

5	10	3	50	1906,35	1
6	1	1	51	2090,48	1
6	1	2	51	1955,56	1
6	1	3	51	1987,30	1
6	2	1	52	2371,43	1
6	2	2	52	2249,21	1
6	2	3	52	2400,00	1
6	3	1	53	2566,67	1
6	3	2	53	2406,35	1
6	3	3	53	2428,57	1
6	4	1	54	1471,43	1
6	4	2	54	1682,54	1
6	4	3	54	1399,81	1
6	5	1	55	2517,46	1
6	5	2	55	2606,35	1
6	5	3	55	2749,21	1
6	6	1	56	687,30	1
6	6	2	56	649,21	1
6	6	3	56	553,97	1
6	7	1	57	903,17	1
6	7	2	57	923,81	1
6	7	3	57	996,83	1
6	8	1	58	2312,70	1
6	8	2	58	2230,16	1
6	8	3	58	2203,17	1
6	9	1	59	1558,73	1
6	9	2	59	1684,13	1
6	9	3	59	1736,51	1
6	10	1	60	1864,29	1
6	10	2	60	1892,06	1
6	10	3	60	1836,51	1
7	1	1	61	1533,33	1
7	1	2	61	1185,71	1
7	1	3	61	951,59	1
7	2	1	62	2057,14	1
7	2	2	62	2312,70	1
7	2	3	62	2083,17	1
7	3	1	63	1884,13	1
7	3	2	63	1831,75	1
7	3	3	63	1820,63	1
7	4	1	64	1041,27	1
7	4	2	64	1109,52	1
7	4	3	64	963,49	1
7	5	1	65	2058,73	1
7	5	2	65	2138,10	1
7	5	3	65	2114,29	1
7	6	1	66	1234,92	1
7	6	2	66	1157,14	1
7	6	3	66	1177,78	1
7	7	1	67	1048,41	1

7	7	2	67	1044,44	1
7	7	3	67	1028,57	1
7	8	1	68	852,38	1
7	8	2	68	766,67	1
7	8	3	68	814,29	1
7	9	1	69	1496,83	1
7	9	2	69	1687,30	1
7	9	3	69	1580,95	1
7	10	1	70	957,14	1
7	10	2	70	828,57	1
7	10	3	70	1006,35	1
8	1	1	71	2080,16	1
8	1	2	71	2094,44	1
8	1	3	71	2302,38	1
8	2	1	72	2361,11	1
8	2	2	72	2508,73	1
8	2	3	72	2342,06	1
8	3	1	73	3357,94	1
8	3	2	73	3365,87	1
8	3	3	73	3453,17	1
8	4	1	74	1916,67	1
8	4	2	74	1992,86	1
8	4	3	74	2119,84	1
8	5	1	75	2476,98	1
8	5	2	75	2610,32	1
8	5	3	75	2596,03	1
8	6	1	76	2723,81	1
8	6	2	76	2794,44	1
8	6	3	76	2653,17	1
8	7	1	77	2080,16	1
8	7	2	77	2167,46	1
8	7	3	77	1865,87	1
8	8	1	78	1740,48	1
8	8	2	78	1799,21	1
8	8	3	78	1715,08	1
8	9	1	79	1913,49	1
8	9	2	79	1918,25	1
8	9	3	79	1707,14	1
8	10	1	80	2357,94	1
8	10	2	80	2332,54	1
8	10	3	80	2429,37	1
9	1	1	81	1842,06	1
9	1	2	81	1913,49	1
9	1	3	81	1946,83	1
9	2	1	82	2159,52	1
9	2	2	82	2118,25	1
9	2	3	82	2803,97	1
9	3	1	83	3032,54	1
9	3	2	83	3215,08	1
9	3	3	83	3256,35	1

9	4	1	84	2289,68	1
9	4	2	84	2350,00	1
9	4	3	84	2121,43	1
9	5	1	85	2278,57	1
9	5	2	85	2302,38	1
9	5	3	85	2302,38	1
9	6	1	86	2429,37	1
9	6	2	86	2500,79	1
9	6	3	86	2508,73	1
9	7	1	87	1810,32	1
9	7	2	87	1745,24	1
9	7	3	87	1773,81	1
9	8	1	88	2183,33	1
9	8	2	88	2056,35	1
9	8	3	88	2297,62	1
9	9	1	89	1389,68	1
9	9	2	89	1207,14	1
9	9	3	89	1191,27	1
9	10	1	90	1619,84	1
9	10	2	90	1675,40	1
9	10	3	90	1659,52	1
10	1	1	91	2032,54	1
10	1	2	91	1857,94	1
10	1	3	91	2103,97	1
10	2	1	92	3350,00	1
10	2	2	92	2984,92	1
10	2	3	92	3294,44	1
10	3	1	93	2826,19	1
10	3	2	93	2889,68	1
10	3	3	93	2215,08	1
10	4	1	94	1969,05	1
10	4	2	94	2010,32	1
10	4	3	94	1897,62	1
10	5	1	95	2318,25	1
10	5	2	95	2426,19	1
10	5	3	95	2326,19	1
10	6	1	96	2770,63	1
10	6	2	96	2799,21	1
10	6	3	96	2762,70	1
10	7	1	97	2659,52	1
10	7	2	97	2919,84	1
10	7	3	97	3135,71	1
10	8	1	98	2484,92	1
10	8	2	98	2513,49	1
10	8	3	98	2635,71	1
10	9	1	99	1651,59	1
10	9	2	99	1683,33	1
10	9	3	99	1715,08	1
10	10	1	100	2421,43	1
10	10	2	100	1964,29	1

10	10	3	100	2357,94	1
1	1	1	1	613,49	2
1	1	2	1	588,10	2
1	1	3	1	596,03	2
1	2	1	2	1860,32	2
1	2	2	2	2241,27	2
1	2	3	2	1693,65	2
1	3	1	3	2233,33	2
1	3	2	3	2122,22	2
1	3	3	3	2077,78	2
1	4	1	4	971,43	2
1	4	2	4	812,70	2
1	4	3	4	868,25	2
1	5	1	5	2233,33	2
1	5	2	5	2225,40	2
1	5	3	5	2239,68	2
1	6	1	6	1423,81	2
1	6	2	6	1762,70	2
1	6	3	6	1741,27	2
1	7	1	7	1257,14	2
1	7	2	7	1582,54	2
1	7	3	7	1511,11	2
1	8	1	8	1376,19	2
1	8	2	8	1193,65	2
1	8	3	8	1352,38	2
1	9	1	9	1138,10	2
1	9	2	9	1196,83	2
1	9	3	9	1223,81	2
1	10	1	10	1265,08	2
1	10	2	10	987,30	2
1	10	3	10	1116,51	2
2	1	1	11	1634,13	2
2	1	2	11	1784,13	2
2	1	3	11	1884,13	2
2	2	1	12	892,06	2
2	2	2	12	827,62	2
2	2	3	12	793,17	2
2	3	1	13	3106,35	2
2	3	2	13	2892,06	2
2	3	3	13	2853,97	2
2	4	1	14	1955,56	2
2	4	2	14	1790,48	2
2	4	3	14	1741,27	2
2	5	1	15	2431,75	2
2	5	2	15	2580,95	2
2	5	3	15	2574,60	2
2	6	1	16	2638,10	2
2	6	2	16	2360,32	2
2	6	3	16	2655,56	2
2	7	1	17	1915,87	2

2	7	2	17	1907,94	2
2	7	3	17	1941,27	2
2	8	1	18	2630,16	2
2	8	2	18	2628,57	2
2	8	3	18	2209,52	2
2	9	1	19	2407,94	2
2	9	2	19	2574,60	2
2	9	3	19	2387,30	2
2	10	1	20	2890,48	2
2	10	2	20	2839,68	2
2	10	3	20	2841,27	2
3	1	1	21	1590,48	2
3	1	2	21	1360,32	2
3	1	3	21	1528,57	2
3	2	1	22	2458,73	2
3	2	2	22	2280,95	2
3	2	3	22	2419,84	2
3	3	1	23	603,97	2
3	3	2	23	629,37	2
3	3	3	23	637,30	2
3	4	1	24	1511,11	2
3	4	2	24	1784,13	2
3	4	3	24	1598,41	2
3	5	1	25	1907,94	2
3	5	2	25	1788,89	2
3	5	3	25	2026,98	2
3	6	1	26	2733,33	2
3	6	2	26	2844,44	2
3	6	3	26	2850,79	2
3	7	1	27	1733,33	2
3	7	2	27	1487,30	2
3	7	3	27	1873,02	2
3	8	1	28	2085,71	2
3	8	2	28	2011,11	2
3	8	3	28	2093,65	2
3	9	1	29	1280,95	2
3	9	2	29	931,75	2
3	9	3	29	1661,90	2
3	10	1	30	1953,97	2
3	10	2	30	1896,83	2
3	10	3	30	2092,06	2
4	1	1	31	1630,16	2
4	1	2	31	1488,49	2
4	1	3	31	1582,54	2
4	2	1	32	2685,71	2
4	2	2	32	2701,59	2
4	2	3	32	2217,46	2
4	3	1	33	2550,79	2
4	3	2	33	2271,59	2
4	3	3	33	2336,51	2

4	4	1	34	685,71	2
4	4	2	34	588,10	2
4	4	3	34	633,17	2
4	5	1	35	812,70	2
4	5	2	35	920,63	2
4	5	3	35	738,89	2
4	6	1	36	1939,68	2
4	6	2	36	2061,90	2
4	6	3	36	2026,98	2
4	7	1	37	1191,75	2
4	7	2	37	1312,70	2
4	7	3	37	1279,37	2
4	8	1	38	904,76	2
4	8	2	38	1019,05	2
4	8	3	38	1011,11	2
4	9	1	39	1084,13	2
4	9	2	39	1011,11	2
4	9	3	39	955,56	2
4	10	1	40	1084,13	2
4	10	2	40	1084,13	2
4	10	3	40	933,33	2
5	1	1	41	1903,17	2
5	1	2	41	1907,94	2
5	1	3	41	1894,29	2
5	2	1	42	1760,32	2
5	2	2	42	1653,97	2
5	2	3	42	1707,14	2
5	3	1	43	1830,16	2
5	3	2	43	1701,59	2
5	3	3	43	2447,62	2
5	4	1	44	682,54	2
5	4	2	44	1122,22	2
5	4	3	44	638,10	2
5	5	1	45	298,41	2
5	5	2	45	253,97	2
5	5	3	45	298,41	2
5	6	1	46	1669,84	2
5	6	2	46	1558,73	2
5	6	3	46	1574,60	2
5	7	1	47	1403,97	2
5	7	2	47	1487,30	2
5	7	3	47	1320,63	2
5	8	1	48	987,30	2
5	8	2	48	1249,21	2
5	8	3	48	1326,98	2
5	9	1	49	1200,00	2
5	9	2	49	1314,29	2
5	9	3	49	1312,70	2
5	10	1	50	1039,68	2
5	10	2	50	1015,87	2

5	10	3	50	917,46	2
6	1	1	51	1304,76	2
6	1	2	51	1220,63	2
6	1	3	51	1288,89	2
6	2	1	52	2161,90	2
6	2	2	52	2114,29	2
6	2	3	52	1946,03	2
6	3	1	53	2455,56	2
6	3	2	53	2282,54	2
6	3	3	53	2207,94	2
6	4	1	54	1249,21	2
6	4	2	54	1457,14	2
6	4	3	54	1257,14	2
6	5	1	55	1560,32	2
6	5	2	55	1631,75	2
6	5	3	55	1795,24	2
6	6	1	56	383,33	2
6	6	2	56	385,32	2
6	6	3	56	387,30	2
6	7	1	57	1225,40	2
6	7	2	57	1185,71	2
6	7	3	57	1138,10	2
6	8	1	58	2150,79	2
6	8	2	58	2103,97	2
6	8	3	58	2052,38	2
6	9	1	59	1383,49	2
6	9	2	59	1433,33	2
6	9	3	59	1526,98	2
6	10	1	60	1233,33	2
6	10	2	60	1233,33	2
6	10	3	60	1169,84	2
7	1	1	61	1725,40	2
7	1	2	61	1415,87	2
7	1	3	61	1098,41	2
7	2	1	62	2269,84	2
7	2	2	62	2574,60	2
7	2	3	62	2363,49	2
7	3	1	63	1201,59	2
7	3	2	63	1582,54	2
7	3	3	63	1312,70	2
7	4	1	64	1336,51	2
7	4	2	64	1463,49	2
7	4	3	64	1219,05	2
7	5	1	65	1225,40	2
7	5	2	65	1312,70	2
7	5	3	65	1126,98	2
7	6	1	66	1046,83	2
7	6	2	66	939,68	2
7	6	3	66	1058,73	2
7	7	1	67	925,24	2

7	7	2	67	931,75	2
7	7	3	67	933,17	2
7	8	1	68	1200,00	2
7	8	2	68	1058,73	2
7	8	3	68	909,52	2
7	9	1	69	1204,76	2
7	9	2	69	1471,43	2
7	9	3	69	1352,38	2
7	10	1	70	844,44	2
7	10	2	70	1054,76	2
7	10	3	70	1257,14	2
8	1	1	71	1818,89	2
8	1	2	71	1860,16	2
8	1	3	71	1901,43	2
8	2	1	72	2925,24	2
8	2	2	72	3025,24	2
8	2	3	72	2837,94	2
8	3	1	73	2887,14	2
8	3	2	73	2703,02	2
8	3	3	73	2766,51	2
8	4	1	74	1742,70	2
8	4	2	74	1861,75	2
8	4	3	74	1949,05	2
8	5	1	75	1949,05	2
8	5	2	75	2488,73	2
8	5	3	75	2574,44	2
8	6	1	76	2621,67	2
8	6	2	76	2639,52	2
8	6	3	76	2591,90	2
8	7	1	77	2149,44	2
8	7	2	77	2639,52	2
8	7	3	77	2347,46	2
8	8	1	78	1552,22	2
8	8	2	78	1425,24	2
8	8	3	78	1520,48	2
8	9	1	79	2099,84	2
8	9	2	79	2488,73	2
8	9	3	79	2139,52	2
8	10	1	80	1941,11	2
8	10	2	80	2091,90	2
8	10	3	80	2155,40	2
9	1	1	81	1623,65	2
9	1	2	81	1828,41	2
9	1	3	81	1782,38	2
9	2	1	82	3409,37	2
9	2	2	82	3115,71	2
9	2	3	82	2718,89	2
9	3	1	83	2760,56	2
9	3	2	83	2793,49	2
9	3	3	83	3115,71	2

9	4	1	84	2352,22	2
9	4	2	84	2395,08	2
9	4	3	84	2314,13	2
9	5	1	85	2155,40	2
9	5	2	85	1956,98	2
9	5	3	85	2080,00	2
9	6	1	86	2576,03	2
9	6	2	86	2806,19	2
9	6	3	86	2274,44	2
9	7	1	87	1680,69	2
9	7	2	87	1690,12	2
9	7	3	87	1679,21	2
9	8	1	88	2266,51	2
9	8	2	88	2552,22	2
9	8	3	88	2742,70	2
9	9	1	89	1258,57	2
9	9	2	89	1369,68	2
9	9	3	89	1210,95	2
9	10	1	90	1798,25	2
9	10	2	90	2076,03	2
9	10	3	90	1830,00	2
10	1	1	91	2163,33	2
10	1	2	91	1845,87	2
10	1	3	91	1798,25	2
10	2	1	92	2385,56	2
10	2	2	92	2401,43	2
10	2	3	92	2425,24	2
10	3	1	93	2393,49	2
10	3	2	93	3155,40	2
10	3	3	93	2917,30	2
10	4	1	94	2214,13	2
10	4	2	94	2242,70	2
10	4	3	94	2528,41	2
10	5	1	95	2547,46	2
10	5	2	95	2861,75	2
10	5	3	95	2949,05	2
10	6	1	96	2703,02	2
10	6	2	96	2703,02	2
10	6	3	96	2576,03	2
10	7	1	97	2330,00	2
10	7	2	97	2844,29	2
10	7	3	97	2988,73	2
10	8	1	98	2036,35	2
10	8	2	98	2266,51	2
10	8	3	98	2036,35	2
10	9	1	99	1449,05	2
10	9	2	99	1612,54	2
10	9	3	99	1520,48	2
10	10	1	100	2155,40	2
10	10	2	100	1844,29	2

10	10	3	100	1972,86	2
1	1	1	1	747,50	3
1	1	2	1	929,58	3
1	1	3	1	802,50	3
1	2	1	2	4504,58	3
1	2	2	2	4494,58	3
1	2	3	2	4280,83	3
1	3	1	3	3760,42	3
1	3	2	3	3838,33	3
1	3	3	3	3764,17	3
1	4	1	4	1907,08	3
1	4	2	4	2002,92	3
1	4	3	4	1969,38	3
1	5	1	5	3804,58	3
1	5	2	5	3881,67	3
1	5	3	5	4072,92	3
1	6	1	6	3111,67	3
1	6	2	6	3571,67	3
1	6	3	6	3354,17	3
1	7	1	7	2750,42	3
1	7	2	7	2645,00	3
1	7	3	7	2271,25	3
1	8	1	8	3265,00	3
1	8	2	8	3529,17	3
1	8	3	8	3333,75	3
1	9	1	9	2656,25	3
1	9	2	9	2568,33	3
1	9	3	9	2836,67	3
1	10	1	10	2309,58	3
1	10	2	10	1945,42	3
1	10	3	10	2011,25	3
2	1	1	11	3306,25	3
2	1	2	11	3028,33	3
2	1	3	11	3009,17	3
2	2	1	12	1993,33	3
2	2	2	12	1881,67	3
2	2	3	12	1939,17	3
2	3	1	13	2712,08	3
2	3	2	13	3143,33	3
2	3	3	13	3718,33	3
2	4	1	14	3795,00	3
2	4	2	14	3712,08	3
2	4	3	14	3545,83	3
2	5	1	15	2970,83	3
2	5	2	15	2587,50	3
2	5	3	15	2974,17	3
2	6	1	16	5462,92	3
2	6	2	16	5999,17	3
2	6	3	16	5871,67	3
2	7	1	17	2760,00	3

2	7	2	17	2434,17	3
2	7	3	17	2213,75	3
2	8	1	18	4686,25	3
2	8	2	18	4347,92	3
2	8	3	18	4955,00	3
2	9	1	19	3197,08	3
2	9	2	19	3037,92	3
2	9	3	19	3363,75	3
2	10	1	20	4905,00	3
2	10	2	20	5232,50	3
2	10	3	20	5190,42	3
3	1	1	21	5452,92	3
3	1	2	21	5027,92	3
3	1	3	21	4927,50	3
3	2	1	22	3612,92	3
3	2	2	22	4082,50	3
3	2	3	22	3727,92	3
3	3	1	23	1172,50	3
3	3	2	23	1143,75	3
3	3	3	23	1115,00	3
3	4	1	24	2859,17	3
3	4	2	24	2501,25	3
3	4	3	24	2913,33	3
3	5	1	25	4877,92	3
3	5	2	25	4846,25	3
3	5	3	25	4597,08	3
3	6	1	26	3028,33	3
3	6	2	26	3287,08	3
3	6	3	26	3227,08	3
3	7	1	27	4063,33	3
3	7	2	27	3935,00	3
3	7	3	27	3948,33	3
3	8	1	28	3668,75	3
3	8	2	28	3558,75	3
3	8	3	28	3439,58	3
3	9	1	29	4015,42	3
3	9	2	29	3965,83	3
3	9	3	29	3894,58	3
3	10	1	30	3833,33	3
3	10	2	30	3881,25	3
3	10	3	30	3761,67	3
4	1	1	31	3948,33	3
4	1	2	31	3536,25	3
4	1	3	31	3472,50	3
4	2	1	32	3520,42	3
4	2	2	32	3632,08	3
4	2	3	32	3536,25	3
4	3	1	33	4964,17	3
4	3	2	33	4727,92	3
4	3	3	33	4827,08	3

4	4	1	34	532,08	3
4	4	2	34	515,00	3
4	4	3	34	512,92	3
4	5	1	35	2137,08	3
4	5	2	35	2185,00	3
4	5	3	35	2062,08	3
4	6	1	36	4983,33	3
4	6	2	36	4913,33	3
4	6	3	36	4459,58	3
4	7	1	37	2769,58	3
4	7	2	37	3018,75	3
4	7	3	37	2887,50	3
4	8	1	38	3018,75	3
4	8	2	38	2809,58	3
4	8	3	38	2740,83	3
4	9	1	39	3747,08	3
4	9	2	39	3769,58	3
4	9	3	39	3418,75	3
4	10	1	40	3727,92	3
4	10	2	40	3287,08	3
4	10	3	40	3593,75	3
5	1	1	41	4092,08	3
5	1	2	41	3986,67	3
5	1	3	41	4538,33	3
5	2	1	42	3737,50	3
5	2	2	42	3814,17	3
5	2	3	42	4331,67	3
5	3	1	43	4600,00	3
5	3	2	43	4398,75	3
5	3	3	43	4916,25	3
5	4	1	44	3162,50	3
5	4	2	44	3380,00	3
5	4	3	44	3016,25	3
5	5	1	45	546,25	3
5	5	2	45	555,83	3
5	5	3	45	594,17	3
5	6	1	46	4552,08	3
5	6	2	46	4288,54	3
5	6	3	46	3680,00	3
5	7	1	47	3296,67	3
5	7	2	47	3181,67	3
5	7	3	47	3063,33	3
5	8	1	48	4223,33	3
5	8	2	48	4782,08	3
5	8	3	48	3958,33	3
5	9	1	49	3181,67	3
5	9	2	49	3210,42	3
5	9	3	49	2635,42	3
5	10	1	50	4317,29	3
5	10	2	50	4235,83	3

5	10	3	50	4168,75	3
6	1	1	51	3838,13	3
6	1	2	51	3622,50	3
6	1	3	51	3948,33	3
6	2	1	52	5325,42	3
6	2	2	52	5587,08	3
6	2	3	52	5386,25	3
6	3	1	53	4507,50	3
6	3	2	53	4600,00	3
6	3	3	53	4296,67	3
6	4	1	54	2405,42	3
6	4	2	54	1887,92	3
6	4	3	54	1951,25	3
6	5	1	55	3593,75	3
6	5	2	55	3028,33	3
6	5	3	55	3162,50	3
6	6	1	56	760,00	3
6	6	2	56	621,67	3
6	6	3	56	754,58	3
6	7	1	57	2587,50	3
6	7	2	57	2226,67	3
6	7	3	57	2491,67	3
6	8	1	58	3545,83	3
6	8	2	58	3603,33	3
6	8	3	58	3411,67	3
6	9	1	59	3788,75	3
6	9	2	59	3871,67	3
6	9	3	59	3859,58	3
6	10	1	60	4628,75	3
6	10	2	60	4580,83	3
6	10	3	60	4437,08	3
7	1	1	61	4741,25	3
7	1	2	61	5079,17	3
7	1	3	61	5066,67	3
7	2	1	62	3028,33	3
7	2	2	62	2956,25	3
7	2	3	62	2865,83	3
7	3	1	63	3152,92	3
7	3	2	63	3568,33	3
7	3	3	63	3625,83	3
7	4	1	64	1939,17	3
7	4	2	64	2405,42	3
7	4	3	64	1672,29	3
7	5	1	65	4620,83	3
7	5	2	65	4935,42	3
7	5	3	65	4894,17	3
7	6	1	66	2049,17	3
7	6	2	66	1792,08	3
7	6	3	66	1427,92	3
7	7	1	67	1377,92	3

7	7	2	67	1260,83	3
7	7	3	67	1397,92	3
7	8	1	68	2645,00	3
7	8	2	68	2429,17	3
7	8	3	68	2387,08	3
7	9	1	69	2204,17	3
7	9	2	69	2582,71	3
7	9	3	69	2817,50	3
7	10	1	70	3517,08	3
7	10	2	70	3929,17	3
7	10	3	70	3918,75	3
8	1	1	71	4887,92	3
8	1	2	71	5322,92	3
8	1	3	71	5581,25	3
8	2	1	72	3252,50	3
8	2	2	72	3476,25	3
8	2	3	72	3308,33	3
8	3	1	73	4540,00	3
8	3	2	73	4680,42	3
8	3	3	73	4746,04	3
8	4	1	74	3179,17	3
8	4	2	74	3281,25	3
8	4	3	74	2882,08	3
8	5	1	75	4832,29	3
8	5	2	75	5188,33	3
8	5	3	75	5466,25	3
8	6	1	76	5115,00	3
8	6	2	76	5341,67	3
8	6	3	76	4802,08	3
8	7	1	77	3434,58	3
8	7	2	77	3083,33	3
8	7	3	77	2782,92	3
8	8	1	78	2121,67	3
8	8	2	78	1728,75	3
8	8	3	78	1613,75	3
8	9	1	79	4214,17	3
8	9	2	79	4278,33	3
8	9	3	79	4479,17	3
8	10	1	80	4642,08	3
8	10	2	80	4776,25	3
8	10	3	80	4841,67	3
9	1	1	81	6070,00	3
9	1	2	81	5853,33	3
9	1	3	81	5770,00	3
9	2	1	82	3712,50	3
9	2	2	82	3351,67	3
9	2	3	82	3770,42	3
9	3	1	83	3405,83	3
9	3	2	83	3267,92	3
9	3	3	83	3099,17	3

9	4	1	84	4268,33	3
9	4	2	84	4205,00	3
9	4	3	84	4310,00	3
9	5	1	85	4664,58	3
9	5	2	85	4920,00	3
9	5	3	85	4647,92	3
9	6	1	86	4760,42	3
9	6	2	86	5226,67	3
9	6	3	86	4819,38	3
9	7	1	87	6041,25	3
9	7	2	87	5635,83	3
9	7	3	87	5780,00	3
9	8	1	88	3699,58	3
9	8	2	88	4047,92	3
9	8	3	88	3549,58	3
9	9	1	89	2447,50	3
9	9	2	89	2452,50	3
9	9	3	89	2505,00	3
9	10	1	90	3089,58	3
9	10	2	90	3089,58	3
9	10	3	90	3112,08	3
10	1	1	91	3894,58	3
10	1	2	91	3737,92	3
10	1	3	91	3714,58	3
10	2	1	92	4651,67	3
10	2	2	92	4416,67	3
10	2	3	92	4344,17	3
10	3	1	93	4237,08	3
10	3	2	93	3885,00	3
10	3	3	93	3725,00	3
10	4	1	94	4038,33	3
10	4	2	94	3677,50	3
10	4	3	94	3875,42	3
10	5	1	95	5600,42	3
10	5	2	95	5191,67	3
10	5	3	95	5367,92	3
10	6	1	96	7317,08	3
10	6	2	96	7028,75	3
10	6	3	96	6772,08	3
10	7	1	97	5657,92	3
10	7	2	97	5365,63	3
10	7	3	97	5540,42	3
10	8	1	98	4258,75	3
10	8	2	98	4415,42	3
10	8	3	98	4415,42	3
10	9	1	99	4191,67	3
10	9	2	99	4322,92	3
10	9	3	99	4257,92	3
10	10	1	100	2857,08	3
10	10	2	100	2690,00	3

10	10	3	100	2782,92	3
1	1	1	1	695,83	4
1	1	2	1	718,75	4
1	1	3	1	720,00	4
1	2	1	2	3940,63	4
1	2	2	2	4207,08	4
1	2	3	2	3667,92	4
1	3	1	3	3112,08	4
1	3	2	3	3657,50	4
1	3	3	3	3421,25	4
1	4	1	4	1745,83	4
1	4	2	4	2031,67	4
1	4	3	4	2116,25	4
1	5	1	5	3183,33	4
1	5	2	5	3699,17	4
1	5	3	5	3539,17	4
1	6	1	6	2827,08	4
1	6	2	6	2759,58	4
1	6	3	6	3160,83	4
1	7	1	7	2807,92	4
1	7	2	7	2955,00	4
1	7	3	7	2807,92	4
1	8	1	8	2506,67	4
1	8	2	8	2188,33	4
1	8	3	8	2937,92	4
1	9	1	9	2765,00	4
1	9	2	9	2922,92	4
1	9	3	9	3047,50	4
1	10	1	10	2705,83	4
1	10	2	10	2568,33	4
1	10	3	10	2635,42	4
2	1	1	11	3274,17	4
2	1	2	11	3018,75	4
2	1	3	11	3071,46	4
2	2	1	12	814,58	4
2	2	2	12	881,67	4
2	2	3	12	849,58	4
2	3	1	13	1782,50	4
2	3	2	13	1663,33	4
2	3	3	13	1226,67	4
2	4	1	14	2271,25	4
2	4	2	14	3267,92	4
2	4	3	14	2271,25	4
2	5	1	15	2510,83	4
2	5	2	15	2338,33	4
2	5	3	15	2766,25	4
2	6	1	16	3739,58	4
2	6	2	16	4262,50	4
2	6	3	16	3908,75	4
2	7	1	17	3416,25	4

2	7	2	17	3247,50	4
2	7	3	17	3545,83	4
2	8	1	18	3858,75	4
2	8	2	18	3727,92	4
2	8	3	18	4000,00	4
2	9	1	19	2891,67	4
2	9	2	19	2774,58	4
2	9	3	19	3025,42	4
2	10	1	20	4252,08	4
2	10	2	20	4047,50	4
2	10	3	20	4465,83	4
3	1	1	21	4494,58	4
3	1	2	21	3440,42	4
3	1	3	21	3545,83	4
3	2	1	22	3697,92	4
3	2	2	22	4301,25	4
3	2	3	22	3865,42	4
3	3	1	23	888,33	4
3	3	2	23	853,33	4
3	3	3	23	1038,75	4
3	4	1	24	2543,75	4
3	4	2	24	2365,83	4
3	4	3	24	3825,42	4
3	5	1	25	3497,92	4
3	5	2	25	2903,75	4
3	5	3	25	2855,83	4
3	6	1	26	3629,58	4
3	6	2	26	3473,75	4
3	6	3	26	3893,33	4
3	7	1	27	2165,83	4
3	7	2	27	2514,17	4
3	7	3	27	2641,67	4
3	8	1	28	3005,00	4
3	8	2	28	2610,83	4
3	8	3	28	3089,58	4
3	9	1	29	1590,83	4
3	9	2	29	2127,50	4
3	9	3	29	2444,17	4
3	10	1	30	2934,17	4
3	10	2	30	2470,00	4
3	10	3	30	2577,92	4
4	1	1	31	2677,50	4
4	1	2	31	2523,33	4
4	1	3	31	2312,50	4
4	2	1	32	2450,00	4
4	2	2	32	1840,00	4
4	2	3	32	1849,58	4
4	3	1	33	3009,17	4
4	3	2	33	2974,17	4
4	3	3	33	2558,75	4

4	4	1	34	474,58	4
4	4	2	34	474,58	4
4	4	3	34	502,08	4
4	5	1	35	1530,83	4
4	5	2	35	1620,83	4
4	5	3	35	1618,75	4
4	6	1	36	2902,92	4
4	6	2	36	2854,58	4
4	6	3	36	3022,50	4
4	7	1	37	2002,92	4
4	7	2	37	2098,75	4
4	7	3	37	2146,67	4
4	8	1	38	2328,75	4
4	8	2	38	2022,08	4
4	8	3	38	2050,83	4
4	9	1	39	2807,92	4
4	9	2	39	3450,00	4
4	9	3	39	3143,33	4
4	10	1	40	2753,75	4
4	10	2	40	2743,33	4
4	10	3	40	2452,08	4
5	1	1	41	3443,75	4
5	1	2	41	3210,42	4
5	1	3	41	3360,42	4
5	2	1	42	3184,17	4
5	2	2	42	2730,00	4
5	2	3	42	2717,92	4
5	3	1	43	4130,00	4
5	3	2	43	3897,08	4
5	3	3	43	3850,00	4
5	4	1	44	2130,42	4
5	4	2	44	2497,08	4
5	4	3	44	2425,83	4
5	5	1	45	421,25	4
5	5	2	45	460,00	4
5	5	3	45	412,08	4
5	6	1	46	3210,42	4
5	6	2	46	3152,92	4
5	6	3	46	3083,75	4
5	7	1	47	2788,75	4
5	7	2	47	2431,25	4
5	7	3	47	2779,17	4
5	8	1	48	3715,42	4
5	8	2	48	3833,33	4
5	8	3	48	3641,67	4
5	9	1	49	2969,58	4
5	9	2	49	3241,67	4
5	9	3	49	2873,75	4
5	10	1	50	3632,08	4
5	10	2	50	3472,50	4

5	10	3	50	3584,17	4
6	1	1	51	3823,75	4
6	1	2	51	3612,92	4
6	1	3	51	3842,92	4
6	2	1	52	3692,92	4
6	2	2	52	3202,92	4
6	2	3	52	3612,92	4
6	3	1	53	3823,75	4
6	3	2	53	3280,83	4
6	3	3	53	3459,58	4
6	4	1	54	1805,00	4
6	4	2	54	2075,63	4
6	4	3	54	1622,50	4
6	5	1	55	3156,25	4
6	5	2	55	3555,42	4
6	5	3	55	3507,50	4
6	6	1	56	575,00	4
6	6	2	56	545,00	4
6	6	3	56	639,58	4
6	7	1	57	2083,33	4
6	7	2	57	1753,75	4
6	7	3	57	1963,75	4
6	8	1	58	1863,75	4
6	8	2	58	1964,58	4
6	8	3	58	2005,42	4
6	9	1	59	2357,50	4
6	9	2	59	3248,33	4
6	9	3	59	3833,33	4
6	10	1	60	3660,83	4
6	10	2	60	3509,17	4
6	10	3	60	3613,33	4
7	1	1	61	3385,00	4
7	1	2	61	3440,42	4
7	1	3	61	3456,67	4
7	2	1	62	3136,67	4
7	2	2	62	3152,92	4
7	2	3	62	3277,50	4
7	3	1	63	3085,83	4
7	3	2	63	3382,92	4
7	3	3	63	3093,33	4
7	4	1	64	2250,83	4
7	4	2	64	2655,83	4
7	4	3	64	2223,75	4
7	5	1	65	2836,67	4
7	5	2	65	2830,42	4
7	5	3	65	2315,83	4
7	6	1	66	2057,92	4
7	6	2	66	2156,25	4
7	6	3	66	2050,83	4
7	7	1	67	1255,42	4

7	7	2	67	1207,50	4
7	7	3	67	1188,33	4
7	8	1	68	1619,58	4
7	8	2	68	2024,58	4
7	8	3	68	1983,75	4
7	9	1	69	1845,00	4
7	9	2	69	1935,83	4
7	9	3	69	1817,08	4
7	10	1	70	3229,58	4
7	10	2	70	3555,42	4
7	10	3	70	3604,58	4
8	1	1	71	4312,92	4
8	1	2	71	4696,67	4
8	1	3	71	4428,33	4
8	2	1	72	2920,42	4
8	2	2	72	3281,25	4
8	2	3	72	2830,83	4
8	3	1	73	3549,58	4
8	3	2	73	3476,25	4
8	3	3	73	3397,92	4
8	4	1	74	3533,75	4
8	4	2	74	3616,67	4
8	4	3	74	4019,17	4
8	5	1	75	3457,08	4
8	5	2	75	3631,04	4
8	5	3	75	3805,00	4
8	6	1	76	3856,25	4
8	6	2	76	4319,58	4
8	6	3	76	3834,58	4
8	7	1	77	2533,75	4
8	7	2	77	2763,75	4
8	7	3	77	2217,50	4
8	8	1	78	2238,33	4
8	8	2	78	2016,25	4
8	8	3	78	2240,42	4
8	9	1	79	3559,17	4
8	9	2	79	3457,08	4
8	9	3	79	3744,58	4
8	10	1	80	2744,58	4
8	10	2	80	2866,67	4
8	10	3	80	3281,25	4
9	1	1	81	3661,67	4
9	1	2	81	4210,83	4
9	1	3	81	3726,67	4
9	2	1	82	2758,75	4
9	2	2	82	2752,92	4
9	2	3	82	3072,92	4
9	3	1	83	3840,42	4
9	3	2	83	4105,42	4
9	3	3	83	3933,33	4

9	4	1	84	2897,92	4
9	4	2	84	3415,42	4
9	4	3	84	2763,75	4
9	5	1	85	3538,75	4
9	5	2	85	3653,75	4
9	5	3	85	3734,17	4
9	6	1	86	4541,25	4
9	6	2	86	4852,92	4
9	6	3	86	4651,67	4
9	7	1	87	2112,08	4
9	7	2	87	1671,25	4
9	7	3	87	2246,25	4
9	8	1	88	3393,33	4
9	8	2	88	3530,42	4
9	8	3	88	2936,25	4
9	9	1	89	2313,33	4
9	9	2	89	2615,83	4
9	9	3	89	2461,67	4
9	10	1	90	2418,75	4
9	10	2	90	2428,33	4
9	10	3	90	2802,08	4
10	1	1	91	2757,08	4
10	1	2	91	3040,00	4
10	1	3	91	2648,75	4
10	2	1	92	2543,33	4
10	2	2	92	2418,75	4
10	2	3	92	2706,25	4
10	3	1	93	4045,00	4
10	3	2	93	3674,17	4
10	3	3	93	3415,42	4
10	4	1	94	3137,50	4
10	4	2	94	3482,50	4
10	4	3	94	3161,25	4
10	5	1	95	3070,42	4
10	5	2	95	2859,58	4
10	5	3	95	3377,08	4
10	6	1	96	5440,83	4
10	6	2	96	5135,00	4
10	6	3	96	5230,00	4
10	7	1	97	2787,08	4
10	7	2	97	3262,08	4
10	7	3	97	3463,33	4
10	8	1	98	3548,75	4
10	8	2	98	3983,33	4
10	8	3	98	3415,83	4
10	9	1	99	2584,58	4
10	9	2	99	2754,17	4
10	9	3	99	2888,33	4
10	10	1	100	3549,58	4
10	10	2	100	3054,58	4

10	10	3	100	3380,42	4
